# Blood Transcriptional Profiling Reveals Immunological Signatures of Distinct States of Infection of Humans with *Leishmania infantum*

**DOI:** 10.1371/journal.pntd.0005123

**Published:** 2016-11-09

**Authors:** Luiz Gustavo Gardinassi, Gustavo Rocha Garcia, Carlos Henrique Nery Costa, Vladimir Costa Silva, Isabel Kinney Ferreira de Miranda Santos

**Affiliations:** 1 Department of Biochemistry and Immunology, Ribeirão Preto Medical School, University of São Paulo, Ribeirão Preto, São Paulo, Brazil; 2 Department of Community Medicine, Natan Portela Institute for Tropical Diseases, Federal University of Piauí, Teresina, Brazil; Liverpool School of Tropical Medicine, UNITED KINGDOM

## Abstract

Visceral leishmaniasis (VL) can be lethal if untreated; however, the majority of human infections with the etiological agents are asymptomatic. Using Illumina Bead Chip microarray technology, we investigated the patterns of gene expression in blood of active VL patients, asymptomatic infected individuals, patients under remission of VL and controls. Computational analyses based on differential gene expression, gene set enrichment, weighted gene co-expression networks and cell deconvolution generated data demonstrating discriminative transcriptional signatures. VL patients exhibited transcriptional profiles associated with pathways and gene modules reflecting activation of T lymphocytes via MHC class I and type I interferon signaling, as well as an overall down regulation of pathways and gene modules related to myeloid cells, mainly due to differences in the relative proportions of monocytes and neutrophils. Patients under remission of VL presented heterogeneous transcriptional profiles associated with activation of T lymphocytes via MHC class I, type I interferon signaling and cell cycle and, importantly, transcriptional activity correlated with activation of Notch signaling pathway and gene modules that reflected increased proportions of B cells after treatment of disease. Asymptomatic and uninfected individuals presented similar gene expression profiles, nevertheless, asymptomatic individuals exhibited particularities which suggest an efficient regulation of lymphocyte activation and a strong association with a type I interferon response. Of note, we validated a set of target genes by RT-qPCR and demonstrate the robustness of expression data acquired by microarray analysis. In conclusion, this study profiles the immune response during distinct states of infection of humans with *Leishmania infantum* with a novel strategy that indicates the molecular pathways that contribute to the progression of the disease, while also providing insights into transcriptional activity that can drive protective mechanisms.

## Author Summary

Infections of humans with the protozoan parasites *L*. *donvani* and *L*. *infantum* can lead to the development of the disease visceral leishmaniasis, but also to an asymptomatic status. However, the mechanisms that result in these clinical outcomes after infection are poorly understood. In this study, we applied a data-driven approach to obtain insights into the immunological processes linked to the progression of the disease or to protective mechanisms. For this purpose, we evaluated the patterns of expression for genes that code proteins from the entire human genome in the peripheral blood from patients with visceral leishmaniasis, from individuals who remained asymptomatic after infections with *L*. *infantum*, from patients who were recovering from disease after treatment and from uninfected individuals. By employing computational analysis to evaluate the blood transcriptional activity of each group, we identified transcriptional signatures that correlate with previous findings obtained through different analytical methods. Moreover, our analyses uncovered hitherto unidentified molecular pathways and gene networks associated with the transcriptional profiles of individuals recovering from disease or that did not develop symptoms after infection. This suggests that activation of protective responses can be useful targets for the development of new therapies for visceral leishmaniasis.

## Introduction

Infections with the protozoan parasites *Leishmania donovani* or *L*. *infantum (chagasi)* result in clinical outcomes that range from asymptomatic infection to active visceral leishmaniasis (VL). When disease occurs, symptoms often include fever, hepatosplenomegaly, cachexia, pancytopenia and hypergammaglobulinemia [1], while the lethality of VL correlates with severe symptoms such as secondary infections, hemorrhage, liver failure and cardiotoxicity due to treatment [2].

Depressed cellular immunity is considered a hallmark of VL, which is evidenced by the inability of VL patients to develop a positive delayed type hypersensitivity (DTH) in Montenegro skin tests in spite of infection [3], and the absence of IFN-γ in cultures of peripheral blood mononuclear cells stimulated with leishmanial antigens [4]. On the other hand, whole blood assays showed that VL patients do not lack the ability to mount *Leishmania* specific IFN-γ responses [5]. Furthermore, peripheral blood or splenic CD4^+^ T lymphocytes from VL patients produce IFN-γ in response to leishmanial antigens, which is also crucial to limit parasite replication in splenic aspirate cultures [6]. These findings indicate that progression of VL involves other molecular mechanisms besides failures in activation and differentiation of CD4^+^ T lymphocytes. Development and severity of VL have been associated with several pro-inflammatory and immunoregulatory factors such as cytokines [7,8], lipopolysaccharide [9], mannan-binding lectin [10], C reactive protein and patterns of IgG Fc N-glycosylation [8]. In addition, studies addressing features of infected asymptomatic individuals point towards a fine regulation of several immune compartments thought to control parasites without damage to the host [8,11,12]. Thus, particular clinical outcomes after infections with *L*. *infantum* are influenced by complex multi-factorial immunological processes.

Re-circulation between central and peripheral lymphoid organs has a major impact on effective immune responses and infections and inflammation cause cell migration via lymphatic and circulatory systems [13]. During physiological or pathological events in which factors are released systemically, features of peripheral cell re-circulation provide an informative platform to study the human immune system with molecular methods of genomic scale, which have been used to investigate blood transcriptional and immunological profiles during human infections, including parasitic diseases [14–16].

Genome-wide profiling strategies have been employed to evaluate *in vitro* systems of infection with *Leishmania* and *in vivo* models of VL [17–20], while studies in humans are limited to biopsies from patients with cutaneous leishmaniasis [21,22]. We hypothesized that a global overview of gene expression in the peripheral blood of humans presenting with distinct states of infection with *L*. *infantum* could reveal unappreciated immunological features that account for pathological or protective responses. To address this issue, we undertook a series of molecular approaches and functional analyses to uncover the transcriptional activity of the immune response that extend the understanding and provide new insights into the immunobiology of human VL.

## Methods

### Ethics statement and study groups

This study was conducted as per protocols approved by the Research Ethics Committee of the Clinics Hospital of the Ribeirão Preto Medical School—USP (protocol 2347/2012). All the methods were carried out in accordance with approved guidelines. Informed written consent was obtained from all of the participants or their parents or legal guardians. Whole peripheral blood was collected from patients with symptoms of VL admitted to Natan Portella Institute of Tropical Diseases, UFPI, Teresina-PI, Brazil. Diagnosis was confirmed by identification of Leishmania amastigotes in Giemsa-stained smears of bone marrow aspirate, and patients diagnosed with VL received treatment according to Brazilian guidelines [23]. Additionally, whole peripheral blood was collected from a distinct group of VL patients at 2 to 5 months after the beginning of therapy with pentavalent antimonial, which were under remission of the disease (Table 1). Study subjects also included healthy individuals living in the same areas and considered to be asymptomatically infected with *L*. *infantum*, who were identified by a positive delayed type hypersensitivity (DTH) to leishmanial antigens (Table 1). Controls included individuals from different regions of Brazil (Teresina-PI and Ribeirão Preto-SP) who presented a negative DTH to leishmanial antigens (Table 1). The groups did not present significant differences with respect to age (ANOVA *P* value = 0.370) or sex distribution (Chi-square *P* value = 0.4181). Whole peripheral blood samples were stabilised in PAXgene Blood RNA tubes (PreAnalitiX, Hombrechtikon, Switzerland) and stored at -80°C.

**Table 1:** Human sample groups evaluated in this study. Mean values and standard deviations are shown. Hematological features were evaluated before therapy. VL—patients with visceral leishmaniasis; TRT—treated patients under remission of disease; DTH—asymptomatic individuals; CTRL—uninfected controls; WBC—white blood cell; RBC—red blood cell; Hgb—hemoglobin; Hct—hematocrit; N/D—not done.

Groups	Total no.	Age, yr	Males	Females	WBC (mm^3^)	RBC (10^6^ /mm^3^)	Hgb (g/dL)	Hct (%)
VL	8	22.5 (17.5)	7	1	3740 (1108)	3.5 (0.49)	8.6 (1.23)	26.1 (2.57)
TRT	8	23.4 (20.6)	5	3	2452 (296)	3.2 (0.75)	7.6 (2.18)	23.5 (6.14)
DTH	14	32.0 (17.9)	8	6	N/D	N/D	N/D	N/D
CTRL	15	32.4 (14.3)	8	7	N/D	N/D	N/D	N/D

### RNA isolation and hybridization

Isolation and purification of total RNA was performed using the PAXgene Blood RNA Kit (PreAnalytix) according to the manufacturer’s instructions. RNA concentration was verified with NanoDrop 1000 spectrophotometer (NanoDrop Technologies, Wilmington, DE, USA) and the RNA integrity was determined using an Agilent 2100 Bioanalyzer (Agilent Technologies, Foster City, CA, USA). The RNA samples were submitted to microarray hybridization at the Functional Genomics Unit of the Roy J. Carver Biotechnology Center, University of Illinois, Urbana-Champaign, Illinois, USA. All procedures were performed according to the manufacturer’s instructions. Briefly, cRNA amplification and labelling was carried out on 1 ug of total RNA by using an Illumina TotalPrep Amplification kit (Ambion, Austin, TX, USA). The samples were then hybridized onto on Illumina HumanHT-12 v4 Expression BeadChips that were scanned with an Illumina iScan System (Illumina, San Diego, CA, USA). Illumina´s Beadstudio software was used to generate signal intensity values from the scans.

### Basic bioinformatics analyses

Raw data were processed using the R Language and Environment for Statistical Computing (R) 3.2.0 [24] in association with Bioconductor 3.1 [25]. The *lumi* package for R [26] was used to perform quality control, log_2_ transformation and normalization with robust spline normalization (RSN) method. This processing pipeline was based on the comparison and variation of transformation and normalization methods and optimized according to the number of samples, as well as the array technology [27]. Data was filtered to remove unexpressed genes based on detection call p-values computed for each probeset of the > 47,000 probes present on the Illumina HumanHT-12 v4 array and 17,015 probes were retained for further analysis. Probe-level expression data files were deposited at the Gene Expression Omnibus (GEO) repository under accession number GSE77528.

### Differential gene expression and pathway analysis

The patterns of differential gene expression between the study groups were evaluated by generating linear models and moderated *t*-statistic or ANOVA with the package Limma for R [28]. *P* values were adjusted with Benjamini-Hochberg false discovery rate (FDR) correction, whereby differentially expressed probes were identified by a FDR <0.01 and mean fold-difference ≥ 1.5 between VL patients and controls or asymptomatic individuals; or mean fold-difference ≥ 1.3 between patients under remission and VL patients, controls or asymptomatic individuals. Two different cut-offs of fold-differences were chosen in order to avoid over-estimating differentially expressed probes or penalizing particular transcriptional profiles in pathway analyses. Differentially expressed probes were collapsed into genes using the function collapseRows() and “Max-Mean” method [29] from the WGCNA package for R [30]. On the basis of differentially expressed genes (DEGs) between the study groups, a heat map was generated to visualize the resulting hierarchical clustering of expression data performed with Euclidian distance and complete algorithm linkage. DEGs lists were incorporated to the GeneGo MetaCore pathway analysis tool (Thomson Reuters, NY) and used to identify genes that overlap within curated biological processes and pathways at a higher frequency than would normally be expected to occur for a randomly selected set of genes. A FDR <0.05 was used as a threshold to determine whether a process or pathway was statistically represented by DEGs.

### Weighted gene co-expression network analysis

Co-expressed genes across the whole data set (n = 45 samples in the same analysis) were selected using the weighted gene co-expression network analysis (WGCNA) package for R [30]. Log-transformed, normalized expression data were filtered by the 3700 most variant genes. A soft threshold power beta was chosen based on the scale-free topology criterion [31]. Constructed gene networks were then used to identify modules from the topological overlap matrix with the functions cutreeDynamic() and mergeCloseModules() and imported for network visualization into Cytoscape v 3.2.1.

### Gene set enrichment analysis

Gene Set Enrichment Analysis (GSEA) [32] was used to determine significant associations between blood transcriptional patterns of each study group and the modules identified by WGCNA, which were loaded as gene sets. In addition, we also implemented GSEA based on a framework of Blood Transcriptional Modules (BTM) which was previously constructed from over 30,000 human blood transcriptomes derived from more than 500 studies available in public databases [33]. GSEA parameters included weighted enrichment statistic and Signal2Noise metric, with 1,000 permutations.

### Blood cell deconvolution

To estimate relative abundance of cell subsets from whole blood expression profiles we implemented the meanProfile method with the CellMix package for R [34]. We applied this method using previously published signatures for erythroblasts, megakaryocytes, granulocytes, monocytes, NK cells, CD4^+^ T lymphocytes, CD8^+^ T lymphocytes and B lymphocytes [35].

### Quantitative real time-PCR

Reverse Transcription followed by quantitative PCR (RT-qPCR) was performed using Complementary DNA was synthesized starting from 200 ng of RNA using High Capacity cDNA Reverse Transcription Kit (Applied Biosystems, Foster City, CA). SYBR Green real-time amplifications were performed on a Rotor-Gene 6000 instrument (Corbett Life Science, Valencia, CA, USA) using a set of designed primers (S1 Table). Samples were analyzed in duplicate and values obtained by RT-PCR for target genes were normalized to the average of cycling threshold from "housekeeping" genes *ACTB*, *B2M*, *RNA18S5* and *PPIA*. Fold changes were calculated according to the 2^(-ΔΔCt)^ method.

### Statistical analysis

Data analysis was performed with GraphPad Prism V 5.0. One-way ANOVA with Bonferroni´s multiple-comparison test or one-sample *t* test were used to evaluate differences among independent groups. Spearman’s rank correlation was applied to assess nonparametric associations. *P* values less than 0.05 were considered significant.

## Results

### The abundance of transcripts in peripheral blood depends on the state of infection with *Leishmania infantum*

**Fig 1:**
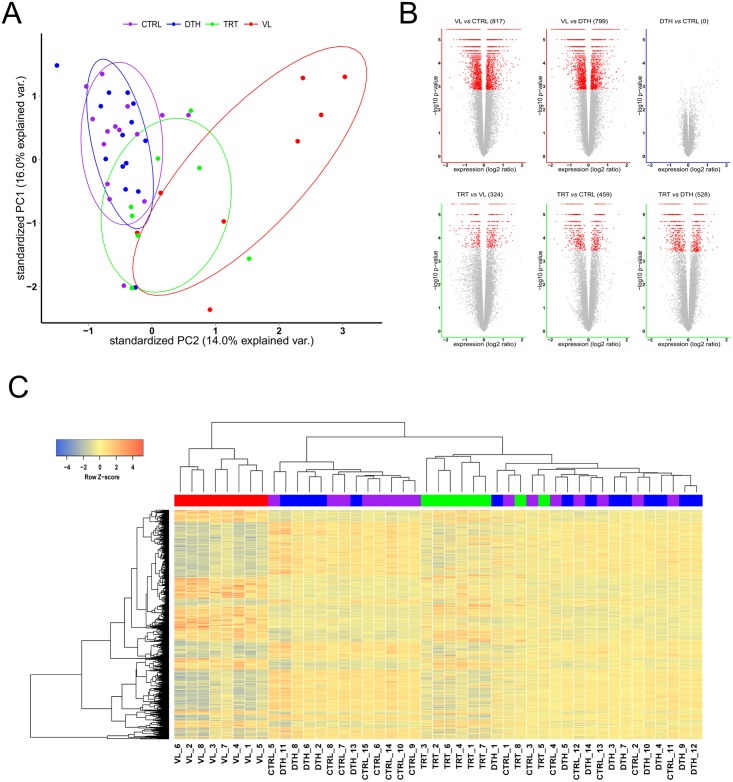
Analysis of blood transcriptomic data from distinct states of infection with *L*. *infantum*. **(A)** Principal component analysis (PCA). Expression data from all 17,105 annotated probe sets were used for this analysis. VL—patients with visceral leishmaniasis (red), DTH—asymptomatic individuals (blue), TRT—treated patients under remission of disease (green) and CTRL—uninfected controls (purple). **(B)** Differential expression analysis between states of infection with *L*. *infantum*. The number of differentially expressed genes (DEGs) are shown, whereby the red dots in volcano plots depict DEGs using a FDR <0.01. **(C)** Unsupervised hierarchical clustering based on expression of DEGs between all states of infection with *L*. *infantum* (ANOVA *P* < 0.001, 2232 genes). The blue to red scale indicates lower to higher expression levels based on a Z-score.

To determine transcriptional signatures associated with distinct states of infection with *L*. *infantum*, we evaluated the patterns of gene expression of whole blood from active VL patients, from patients that received treatment and were considered to be under remission of disease, from healthy individuals that exhibited a positive delayed type hypersensitivity reaction to leishmanial antigens and that were considered to be asymptomatically infected with *L*. *infantum*, and control individuals that exhibited a negative delayed type hypersensitivity to leishmanial antigens and were considered to be uninfected (Table 1). Principal component analysis (PCA) of the 17,105 annotated probe sets (12,491 genes) resulted in a consistent pattern of clustering for half of the VL patients, whereas PCA based on expression data from uninfected controls and asymptomatic individuals indicates similar global transcriptional profiles between those subjects (Fig 1A). In addition, the transcriptional profile of patients under remission of disease exhibited an intermediary pattern of clustering between VL patients and uninfected controls or asymptomatic individuals (Fig 1A). Linear model-based statistical analysis with a FDR < 0.01 identified 817 or 799 differentially expressed genes (DEGs) between VL patients and uninfected controls or asymptomatic individuals, respectively (Fig 1B—upper left and middle panels—and S1 Data). Applying this statistical method, we observed that asymptomatic individuals did not present significant differences in whole blood gene expression when compared to uninfected controls (Fig 1B—upper right panel). Further analysis resulted in 324, 459 or 528 DEGs between expression data from patients under remission and VL patients, uninfected controls or asymptomatic individuals, respectively (Fig 1B—lower panels—and S1 Data).

To evaluate whether transcriptional signatures based on differential expression analysis could segregate subjects from distinct states of infection, we applied an unsupervised hierarchical clustering on expression data from highly significant DEGs between all groups (ANOVA *P* < 0.001, 2232 genes), shown in Fig 1C. The analysis resulted in two main clusters of individuals. The first cluster was comprised only by VL patients (Fig 1C). The second cluster resolved into two sub-clusters; one composed mainly by uninfected controls, but which also contained asymptomatic individuals; and a second sub-cluster formed by patients under remission of disease, asymptomatic individuals and uninfected controls (Fig 1C). Of interest, most patients under remission of disease clustered together into a unique group within this sub-cluster (Fig 1C). Taken together, these results demonstrate that infections with *L*. *infantum* induce significant changes in the abundance of blood transcripts and that the patterns of gene expression depend on the clinical status after infection or activity of the disease.

### Analyses of molecular pathways recruited during infections with *L*. *infantum*

**Fig 2:**
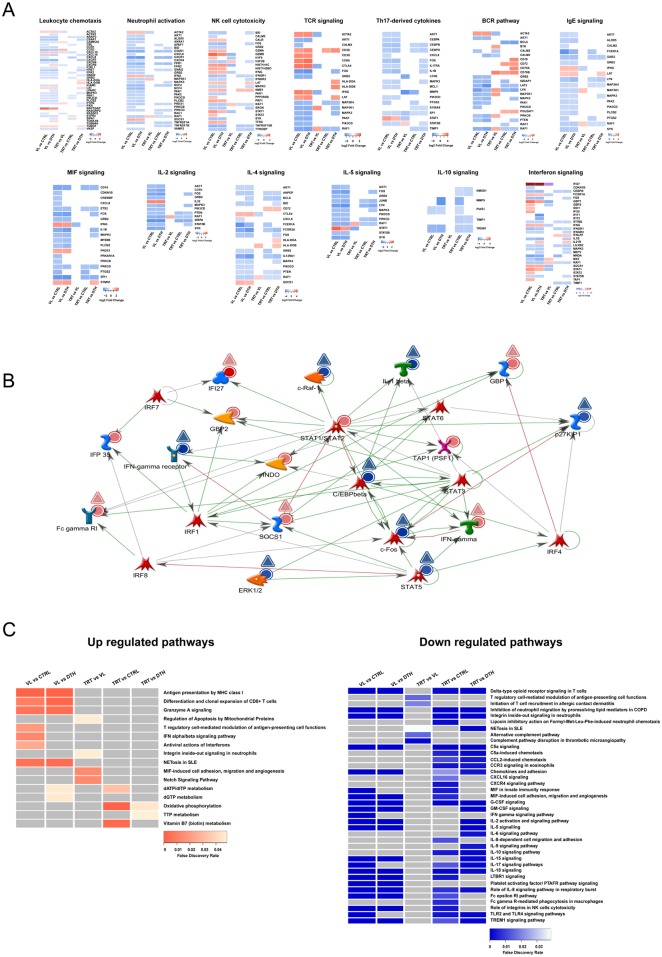
Functional analysis based on differentially expressed genes (DEGs). **(A)** Dynamics of changes in expression of genes enriched for general biological process networks annotated by GeneGO Metacore. **(B)** Interferon signaling process network associated with blood transcriptional profile of VL patients compared to uninfected controls. The network between differentially expressed genes were calculated with the “analyze network” algorithm from the GeneGO MetaCore database. Circles represent DEGs between VL patients and uninfected controls, while triangles represent DEGs between VL patients and asymptomatic individuals. Red and blue depict up-regulated and down-regulated genes, respectively. Green arrows indicate positive interaction, red arrows indicate negative interactions and gray arrows indicate unspecified interactions. See MetaCore website for detailed legend at https://portal.genego.com/legends/MetaCoreQuickReferenceGuide.pdf. **(C)** GeneGO Metacore pathway enrichment analysis of up-regulated or down-regulated genes using a FDR <0.05. Pathways that were not associated with a particular transcriptional profile are depicted in gray.

To understand the biological processes reflected by the identified transcriptional signatures, we used the GeneGO Metacore platform for functional analysis to retrieve the ontology of immunity related genes that were differentially expressed, whereby their expression changed according to each of the comparisons between the states of infection (Fig 2A). Relative to uninfected controls or asymptomatic individuals, the expression of genes annotated into processes such as leukocyte chemotaxis (*CCR1*, *CCR3*, *CXCR1*, *CXCR4*, *CXCL16*, *CXCL8*) or neutrophil activation (*CXCL8*, *FPR1*, *C5AR1*) were down-regulated in VL patients (Fig 2A). On the other hand, up-regulated genes were mainly enriched into network processes such as NK cell cytotoxicity (*GZMA*, *GZMB*, *PRF1*) or TCR signaling (*CD3D*, *CD3G*, *CD8A*, *LAT*) (Fig 2A). Compared to uninfected controls, VL patients exhibited a wide modulation of genes enriched into the interferon signaling network process (*IDO1*, *IFI35*, *IFIT1*, *IFITM2*, *IFNG*, *SOCS1*, *STAT1*, *STAT2*) (Fig 2A and 2B). Yet, compared to asymptomatic individuals, *GBP2*, *IDO1*, *IFI35*, *STAT2*, *TAP1* were not differentially expressed in VL patients, indeed most of the interferon signaling related genes were down-regulated in this group of individuals (Fig 2A and 2B). The majority of genes enriched for the BCR-pathway were down-regulated in VL patients when compared to both uninfected controls and asymptomatic individuals (Fig 2A). However, compared to other clinical-epidemiologic groups analyzed herein, genes enriched for the BCR-pathway (*BTK*, *CD19*, *CD72*, *CD79A*, *CD79B*, *LYN*) were up-regulated in patients under remission of disease (Fig 2A).

To obtain insights into the regulation of canonical pathways reflected by the transcriptional profiles from distinct states of infection with *L*. *infantum*, we analyzed the enrichment of up-regulated or down-regulated DEGs on pathway maps annotated in the GeneGO Metacore database (Fig 2C). Compared to uninfected controls or asymptomatic infected individuals, up-regulated DEGs from VL patients were enriched in pathways such as: "antigen presentation by MHC class I" (S1 Fig), "differentiation and clonal expansion of CD8^+^ T cells" and “Granzyme A signaling” (Fig 2C—left panel). Moreover, only when compared to uninfected controls, up-regulated DEGs from VL patients were enriched in pathways as: "T regulatory cell-mediated modulation of antigen-presenting cell functions", "IFN alpha/beta signaling" and "antiviral actions of interferons" (Fig 2C—left panel). Those results suggest that VL patients exhibit an increased activation of cytotoxic T lymphocytes, which is in agreement with the up-regulation of genes related to TCR signaling (Fig 1A). Moreover, these results also represent the first evidence of an increased activity of type I interferon signaling in humans infected with *L*. *infantum*. Compared to VL patients, up-regulated DEGs from patients under remission were enriched in pathways such as: "integrin inside-out signaling in neutrophils", and "Notch signaling pathway" (Fig 2C—left panel). Moreover, compared to uninfected controls or to asymptomatic individuals, up-regulated DEGs from patients under remission were enriched into metabolic pathways (Fig 2C—left panel).

Compared to uninfected controls or asymptomatic individuals, down-regulated DEGs from VL patients were enriched for pathways such as: "integrin inside-out signaling in neutrophils" (S2 Fig), chemokine and cytokine signaling and immune receptor signaling as shown in the right panel of Fig 2C. Compared to VL patients, down-regulated DEGs identified for patients under remission were significantly enriched into pathways such as: "T regulatory cell-mediated modulation of antigen-presenting cell functions", "initiation of T cell recruitment in allergic contact dermatitis" (Fig 2C—right panel). Furthermore, compared to uninfected controls or asymptomatic individuals, down-regulated DEGs from patients under remission exhibited significant enrichments in pathways previously associated with expression data from VL patients, except for: “lipoxin inhibitory action on formyl-Met-Leu-Phe-induced neutrophil chemotaxis”, “chemokine signaling (CCL2, CCR3, CXCL16, CXCR4)”, “cytokine signaling (IL-6, IL-8, IL-9, IL-10) “and “Fc gamma R-mediated phagocytosis in macrophages” (Fig 2C—right panel).

Overall, those results indicate that upon development of VL, several pathways related to the immune response are subjected to profound perturbations and suggest that the innate immune response is mainly down-regulated. In contrast, treatment might trigger the activation of pathways as Notch signaling (Fig 2C—left panel) or even down-regulate the transcriptional activity of pathways as “T regulatory cell-mediated modulation of antigen presenting cell functions” or “NETosis in SLE” (Fig 2C—right panel).

### Exploring blood transcriptional profiles with modules of co-expressed genes and BTMs

**Fig 3:**
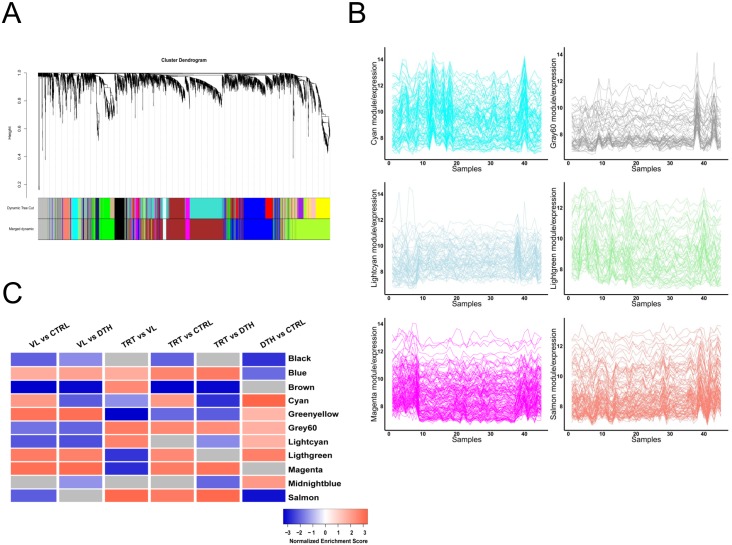
Gene co-expression modules in blood transcriptional profiles identified in distinct states of infection with *L*. *infantum*. **(A)** Cluster dendrogram of highly connected genes was generated with average linkage hierarchical clustering of genes on the basis of topological overlap. Each gene represents a line in the dendogram. Distance between two genes is shown as height on the y-axis. Dynamic tree cutting was used to determine modules. The modules of co-expressed genes were color-coded and further annotated with GeneGO Metacore (see Table 2). **(B)** Linear plots showing fluctuations in blood transcript abundance from cyan, gray60, light-cyan, light-green, magenta and salmon modules (y = log_2_ expression values, x = samples). **(C)** Gene set enrichment analysis of gene modules identified with WGCNA. The blue to red scale indicates negative to positive associations with a determined transcriptional profile based on normalized enrichment scores. Modules that were not associated with a particular profile are depicted in gray.

Although analysis at the level of single genes has been widely used for interpretation of expression data and discovery of biomarkers, the large number of comparisons are permissive to noise and may lack power to detect subtle, but important features of gene expression datasets [32]. Therefore, in order to obtain an additional perspective about the nature of responses reflected by transcriptional profiles from the subjects evaluated herein, we performed a weighted gene co-expression network analysis (WGCNA), which is based on coordinately expressed genes for the identification of gene modules. First, we detected the 3,700 most variable genes from the study population, which included all forty-five samples irrespective of state of infection or treatment. Next, a hierarchical clustering was applied to expression data from those most variable genes, which identified thirteen color-coded co-expression modules (Fig 3A—Merged dynamic). Eleven modules could be annotated with GeneGo Metacore and were enriched in network processes and/or pathway maps described in Table 2; genes composing specific modules are detailed in S2 Data. Demonstrative networks of genes clustered into the cyan module (Type I interferon) or light-green module (antigen presentation by MHC class I) are depicted in S3 Fig.

**Table 2:** Module annotation with GeneGO Metacore pathway analysis.

Modules	Pathway maps and process networks	False Discovery Rate
Black	Translation_Translation initiation	2.17E-14
	Translation_Elongation-Termination	7.78E-10
	Transcription_mRNA processing	2.66E-06
Blue	Immune response_TCR signaling	1.71E-05
	Immune response_Antigen presentation	3.25E-05
	Immune response_Phagosome in antigen presentation	4.81E-03
Brown	Cell adhesion_Leucocyte chemotaxis	5.39E-15
	Cell adhesion_Integrin inside-out signaling in neutrophils	3.27E-12
	Inhibition of neutrophil migration by proresolving lipid mediators in COPD	3.27E-12
Cyan	Inflammation_Interferon signaling	1.67E-23
	Immune response_Antiviral actions of Interferons	3.42E-11
	Immune response_IFN alpha/beta signaling pathway	1.37E-06
Green-yellow	Transcription_Transcription regulation of aminoacid metabolism	1.04E-03
	Cytoskeleton remodeling_TGF. WNT and cytoskeletal remodeling	3.34E-02
	Transcription_Sirtuin6 regulation and functions	4.11E-02
Gray60	Notch signaling pathway	3.92E-02
Light-cyan	Cell adhesion_Leucocyte chemotaxis	1.66E-02
	Cell adhesion_Cadherins	2.24E-02
	Immune response_LTBR1 signaling	2.45E-02
Light-green	Immune response_Antigen presentation by MHC class I	2.38E-03
	Immune response_Antigen presentation	6.75E-03
	Immune response_Phagosome in antigen presentation	4.80E-02
Magenta	Cell cycle_Spindle assembly and chromosome separation	8.12E-04
	Cell cycle_Role of APC in cell cycle regulation	6.43E-03
Midnight blue	Translation_Regulation of initiation	4.46E-02
Salmon	Role of B cells in SLE	1.24E-04

**Fig 4:**
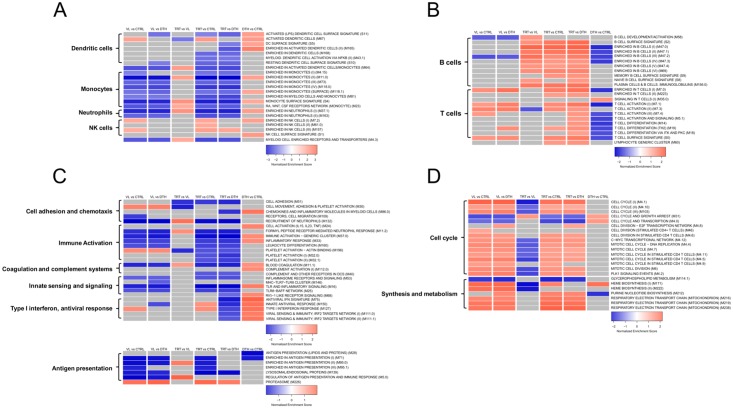
Blood Transcription Modules (BTMs) associated with transcriptomic data from distinct states of infection with *L*. *infantum*. Heat maps illustrate BTMs associated with transcriptional profiles according to each group comparison. Gene set enrichment analysis was used to identify significant associations with modules related to innate immune cells **(A)**; lymphocytes **(B)**; effector and regulatory pathways **(C)**; and cell cycle, synthesis and metabolism **(D)**. The blue to red scale indicates negative to positive associations with a determined transcriptional profile based on normalized enrichment scores. Modules that were not associated with a particular profile are depicted in gray.

WGCNA relies entirely on a data-driven process, which reflects fluctuations in blood transcript abundance measured across an entire population irrespective of state of infection or treatment. Therefore, the transcriptional profiles of the study subjects were graphically represented for individual modules (Fig 3B). Those results demonstrate coordinated expression of genes retained in specific clusters (Fig 3A) and also suggest differential activity of gene modules among distinct clinical-epidemiologic groups (Fig 3B). To further address this question, we conducted gene set enrichment analysis (GSEA) using WGCNA modules as customized gene sets and a FDR <0.05 (Fig 3C). Compared to uninfected controls and asymptomatic individuals, we highlight that the transcriptional profiles of VL patients were associated with a positive regulation of gene modules annotated as: "TCR signaling and antigen presentation" (blue module) and "antigen presentation" (light-green); at the same time, transcriptional profiles of VL patients were associated with a negative regulation of gene modules annotated as: "cell adhesion and neutrophil migration" (brown module), "Notch signaling pathway" (gray60 module) and "cell adhesion and LTBR1 signaling" (light-cyan module) (Fig 3C). Of note, a negative regulation of the module annotated as the "B lymphocyte related module" (salmon) was associated with the transcriptional profile of VL patients only when compared to uninfected controls (Fig 3C). Noteworthy is the fact that a positive regulation of the cyan module (type I interferon, S3A Fig) was also associated with the transcriptional profile of VL patients when compared to uninfected controls, however the same module was negatively regulated in VL patients when compared to asymptomatic individuals (Fig 4C). Overall, compared with VL patients, uninfected controls or asymptomatic individuals, the transcriptional profiles of patients under remission were associated with a positive regulation of modules annotated as: "TCR signaling and antigen presentation" (blue module), "Notch signaling pathway" (Gray60 module) and "B lymphocyte related module" (salmon module) (Fig 3C). Yet, the regulation of several modules associated with transcriptional profiles of patients under remission was dependent on specific comparison with the other clinical-epidemiologic groups (Fig 3C). We also evaluated differences between the transcriptional profiles of asymptomatic individuals in comparison to those of uninfected controls using this same approach. We highlight associations with positive regulations of modules annotated as: "type I interferon signaling" (green-yellow module), "Notch signaling pathway" (gray60 module), "cell adhesion and LTBR1 signaling" (light-cyan module) and "antigen presentation" (light-green module) (Fig 3C); Moreover, asymptomatic individuals exhibited negative regulation of modules as: "TCR signaling and antigen presentation" (blue module) and "B lymphocyte related module" (salmon) (Fig 3C).

Using a different strategy of analysis, we found that results from WGCNA are highly correlated with those from single gene level, capturing significant perturbations of TCR signaling and antigen presentation, as for cell adhesion and neutrophil-related modules in VL patients. Those results also reinforce the fact that treatment of VL patients triggers the activation of Notch signaling pathway and increases the transcriptional activity of B lymphocytes. Of note, using this strategy we identified that, independently of the clinical outcome, infection with *L*. *infantum* induces a transcriptional signature of type I interferon. However, this response exhibits a degree of association with distinct statuses of infection, whereby asymptomatic individuals presented with the strongest associations with activation of this module, followed by VL patients and then by patients under remission of disease.

We also employed GSEA (FDR <0.05) with a previously constructed framework of Blood Transcription Modules (BTMs) [33] to expand the modular analyses obtained by WGCNA and further evaluate the association of transcriptional profiles with distinct status of infection with *L*. *infantum*. Thus, transcriptional profiles of VL patients were associated with a positive regulation of several modules mainly enriched in NK cells (Fig 4A), T lymphocytes (Fig 4B) and type I interferon response (Fig 4C); and cell cycle, synthesis and metabolism (Fig 4D). On the other hand, the transcriptional profiles of VL patients were associated with a negative regulation of several modules related to myeloid cells (Fig 4A), B lymphocytes and effector responses such as cell adhesion and chemotaxis, immune activation with innate sensing and signaling (Fig 4C). Those results are in agreement with data from previous sections, demonstrating up-regulation of genes related to NK cells (Fig 2A) or activation of T lymphocytes (Figs 2 and 3), as well as for an overall down-regulation of the transcriptional activity of the innate immune response (Figs 2 and 3). Compared to VL patients, transcriptional profiles of patients under remission were mainly associated with a positive regulation of modules related to monocytes and neutrophils (Fig 4A), B lymphocytes (Fig 4B), as well as with a few effector pathways such as coagulation and complement systems (Fig 4C). However, relative to uninfected controls and/or asymptomatic individuals, several of those same modules were actually down-regulated, whereby positive associations were found mainly for modules related to NK cells (Fig 4A), both B and T lymphocytes (Fig 4B) and to cell cycle, synthesis and metabolism (Fig 4D). Taken together, those data suggest that treatment induces significant recovery of circulation of neutrophils and monocytes, however not comparable to that seen in healthy individuals. Furthermore, the results suggest that after treatment, there is a more substantial circulation of both B and T lymphocytes, indicating that treatment might function by restoring a balance to adaptive responses (Fig 4B). Additionally, compared to uninfected controls, transcriptional profiles of asymptomatic individuals were associated with a positive regulation of several innate immune cells, including those related to dendritic cells (Fig 4A) whereas modules related to lymphocytes were mainly down-regulated (Fig 4B). Furthermore, several modules related to effector and regulatory pathways were up-regulated in asymptomatic individuals and we highlight the up-regulation of all modules related to type I interferon response, which support the finding that those individuals indeed exhibit the strongest type I interferon response among distinct statuses of infection with *L*. *infantum*. Collectively, those data point to significant differences between blood transcriptional profiles which can reflect molecular mechanisms associated with pathogenic or protective responses during infections with *L*. *infantum*.

Modular analyses are highly informative for capturing differences in immune-related processes during disease, however, recent work demonstrates that gene modules are not independent and are subjected to higher coordinated regulation [36]. In view of that we sought to understand the relationship among the BTMs that were associated to the transcriptional profiles evaluated in this study. Using PCA, we extracted scores from the principal component 1 (PC1) for each of the 101 modules depicted in Fig 4 and performed a hierarchical clustering for coefficients of correlation among PC1 scores, which resulted in 5 main meta-modules (S4 Fig). We highlight meta-module II, which was highly enriched for modules depicting type I interferon signaling, dendritic cells and innate immune activation. Those results corroborate GSEA with BTMs. As an example, asymptomatic individuals indeed exhibited positive associations with several modules involving dendritic cells, innate immune activation and all modules related to type I interferon signaling (Fig 4A and 4C), suggesting a role for dendritic cells in the strong type I interferon signature observed in asymptomatic infection. Indeed, infections with *L*. *major* induce a type I interferon signature in human dendritic cells, which is required for production of IL-12 [37]. In addition, meta-module IV was highly enriched for modules involving B and T lymphocytes, as well as cell cycle (S4 Fig), which is in agreement with the proliferative characteristics of those cells. VL patients exhibited positive associations with modules depicting T lymphocytes and cell cycle, while patients under remission presented up-regulation of modules related to both B and T lymphocytes, as well as cell cycle (Fig 4B and 4D). As expected, those data indicate that BTMs are correlated and support the concept that infections with *L*. *infantum* elicit the coordinated activity of a multi-factorial network of biological processes rather than perturbations in a particular compartment of the immune response.

### Blood cell deconvolution

**Fig 5:**
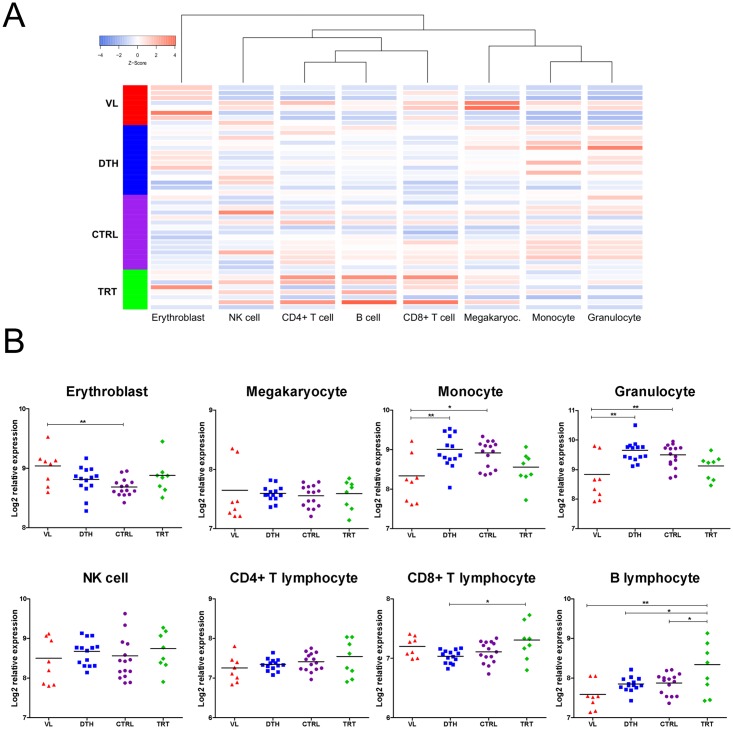
Blood cell deconvolution of transcriptomic data from states of infection with *L*. *infantum*. **(A)** Unsupervised hierarchical clustering of gene expression signatures from erythroblasts, megakaryocytes, granulocytes, monocytes, NK cells, CD4^+^ T lymphocytes, CD8^+^ T lymphocytes and B lymphocytes. Expression values were scaled and displayed as a Z-score, whereby blue indicates decreased expression and red indicates increased expression. **(B)** Comparative analysis of deconvolved expression signatures from blood cells between states of infection with *L*. *infantum*. Statistically significant differences were evaluated with one-way ANOVA followed by Bonferroni´s multiple-comparison test; mean values and significance levels are shown (*, *P* < 0.05 and **, *P* < 0.01).

Whole blood presents a heterogeneous environment composed by numerous distinct, yet interacting cell populations, thus the transcriptional signatures from different states of infection with *L*. *infantum* could represent altered proportions of several cellular subsets. In view of these facts, we undertook a cell deconvolution analysis based on previously published cell signatures [35]. The expression signatures from erythroblasts, megakaryocytes, granulocytes, monocytes, NK cells, CD4^+^ T lymphocytes, CD8^+^ T lymphocytes and B lymphocytes of each study subject are shown in Fig 5A. Compared to uninfected controls, the relative abundance of erythroblasts increased significantly in VL patients (Fig 5B). In contrast, compared to uninfected controls and asymptomatic individuals, the relative abundance of monocytes and granulocytes decreased significantly in VL patients (Fig 5B). Patients under remission exhibited an increase in the relative abundance of CD8^+^ T lymphocytes only when compared to asymptomatic individuals (Fig 5B). However, compared to VL patients, uninfected controls or asymptomatic individuals, the relative abundance of B lymphocytes increased significantly in patients under remission (Fig 5B). Of interest, the relative abundance of megakaryocytes, NK cells and CD4^+^ T lymphocytes did not change among the clinical-epidemiologic groups (Fig 5B). Indeed, those results correlate with those of the modular analyses, which also demonstrate negative associations of myeloid cells with transcriptional profiles from VL patients and patients under remission of disease (Fig 4A), while modules related to B lymphocytes were highly associated with transcriptional profiles of patients under remission of disease (Fig 4B). In view of that, it should be considered that the overall down-regulation of pathways (Fig 2C) or effector and regulatory modules (Figs 3C and 4C) related to the innate immune response observed for VL patients and patients under remission could be driven mainly by decreased proportions of circulating myeloid cells. This decrease, in turn, might be due to entrapment of these cells into the spleen/liver or even be related to defects of the bone marrow and release of cells into the circulation.

### Validation of microarray analyses by quantitative real time-PCR

**Fig 6:**
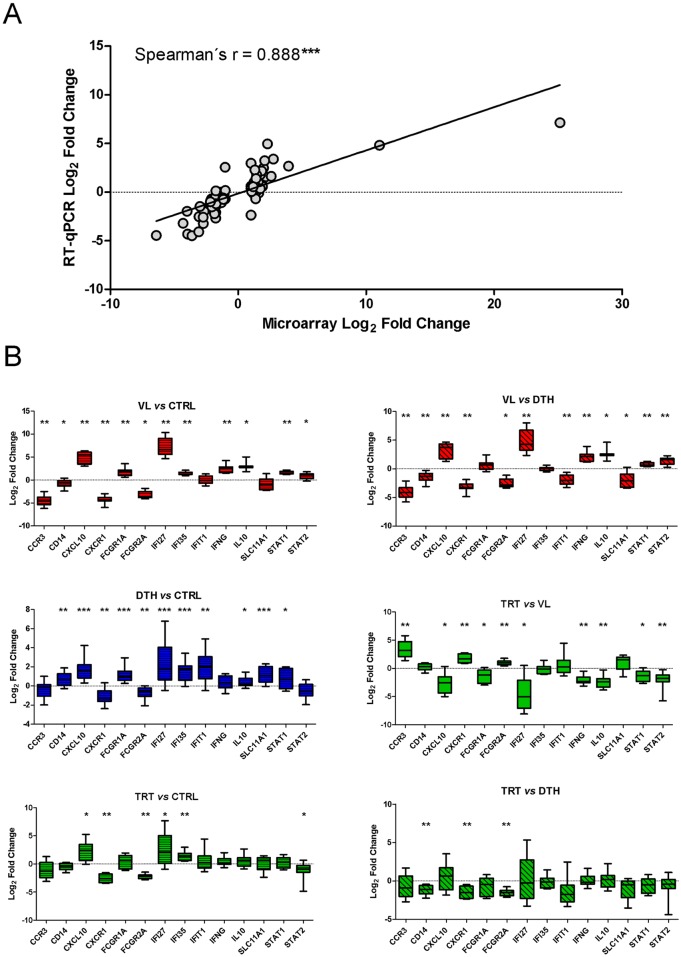
Quantitative Real Time-PCR. **(A)** Changes in relative gene expression are strongly correlated between microarray and RT-qPCR analysis. Each point represents one of the selected genes for each of the comparisons between states of infection with *L*. *infantum*. Correlations between log_2_ fold changes were evaluated by Spearman´s rank correlation. **(B)** Comparative analysis of log_2_ fold changes obtained by RT-qPCR. Statistically significant differences were evaluated with one-way ANOVA followed by Bonferroni´s multiple-comparison test; mean values and significance levels are given (*, *P* < 0.05; **, *P* < 0.01; ***, *P* < 0.001).

To validate the expression obtained by microarray profiling, we also evaluated the expression of a set of genes by RT-qPCR (S1 Table). Relative to the expression of housekeeping genes, fold changes of selected genes were strongly correlated with those obtained with microarray expression profiling (Fig 6A). A detailed analysis of the relative expression of target genes demonstrated that microarray profiling was robust enough to capture significant differences in gene expression of highly modulated blood transcriptional profiles, such as those of VL patients and patients under remission of disease (Fig 6B). Moreover, we found that compared to uninfected controls, asymptomatic individuals exhibited significant modulations on relative expression of the majority of the target genes. Those results support the benefits of combining distinct functional analysis methods in blood transcriptomics and corroborate the findings obtained with WGCNA and GSEA.

## Discussion

The mechanisms that drive progression towards disease or protect individuals from developing symptoms while infected with *Leishmania* parasites remain poorly understood. Using a genome-wide approach to investigate patterns of gene expression from whole blood, we identified transcriptional profiles that shed light on pathways and/or gene expression modules associated with distinct states of human infections with *L*. *infantum*. It is noteworthy that the transcriptional signatures identified in this study discriminated between VL patients from patients under remission of disease and healthy individuals (Fig 1C). Importantly, by assessing the levels of expressions of a set of target genes by RT-qPCR we validated the robustness of the expression data acquired by microarray analysis (Fig 6). Transcriptional signatures from human samples have been shown to be sensitive to factors such as age and sex [38] and sample size [39]. The groups evaluated in this study did not exhibit significant differences in distribution of age or sex. Similar numbers of patients infected with *L*. *braziliensis* and controls were evaluated by pioneering studies that not only identified unique transcriptional signatures, but were also able to recapitulate previously described immunopathological responses in lesions of individuals with cutaneous leishmaniasis [21,22,40]. Of note, samples from patients under remission of disease were collected during distinct time points after the beginning of therapy, which could influence their blood transcriptional profiles. Nonetheless, we were able to identify transcriptional signatures that segregated patients under remission of disease from VL patients before therapy (Fig 1C); concomitantly, relative to asymptomatic individuals or uninfected controls, the majority of patients under remission of disease exhibited strong correlations of levels of expression for DEGs (Fig 1C). Furthermore, linear model-based statistical analysis and adjusted *P* values (FDR) did not detect significant differences between the transcriptional profiles of asymptomatic individuals and uninfected controls (Fig 1B and 1C). However, uncorrected *P* values retrieved 620 differentially expressed probes (S1 Data), which included probes for genes shown to be differentially expressed by RT-qPCR (Fig 6). These results demonstrate that, compared to uninfected controls, asymptomatic individuals present only a subset of differentially expressed genes, whereby the large number of comparisons between 17,105 probes [12,491 genes) can lead to a type II statistical error and inflate the rate of false negatives [41]. To overcome this issue, we conducted distinct approaches with the ability to estimate the differences between the transcriptional profiles of asymptomatic individuals and uninfected controls; the combination of distinct functional analyses and common features retrieved by them support the robustness of the immunological signatures identified for distinct states of infections with *L*. *infantum*. Therefore, we propose that in-depth analysis of transcriptional profiles from such populations, as well as longitudinal studies including patients followed throughout treatment can be useful for the prospection of new biomarkers of VL or asymptomatic infection, as well as for the prognosis after treatment and remission of disease.

Previous analyses demonstrated that the *in vitro* infection of monocyte-derived macrophages (MDM) and dendritic cells (MDC) with *Leishmania* elicits both species and cell-specific expression signatures [17]. Moreover, macrophage cultures infected with species of *L*. *donovani* complex exhibit an overall suppression of gene expression, suggesting a failure of proper macrophage activation [18,42]. However, the regulation of gene expression in MDM infected with *L*. *chagasi* was significantly impacted by the co-culture with autologous *Leishmania-naïve* T cells [18], suggesting that the inflammatory milieu of complex microenvironments such as the infection foci and peripheral blood influence the transcriptional programs of immune cells. Indeed, gene expression profiling of liver-resident macrophages (Kuppfer cells) from mice infected with *L*. *donovani* identified a key transcriptomic network centered around the retinoid X receptor alpha, which was only active in bystander uninfected Kupffer cells exposed to the inflammatory factors in infected livers [19]. As observed for experimental VL [43], our study supports the view of compartmentalized responses, i.e., the dynamics of dominant pathways in specific cells of the spleen, liver, bone marrow and peripheral blood might be differentially associated with pro-inflammatory or regulatory processes during the course of the infection. For instance, there are strong evidences that IL-10 plays a role in the suppression of the response in the spleen of VL patients [44,45], but, despite increased serum concentrations or production of IL-10 in whole blood assays [8,46] and elevated expression of IL-10 found herein by RT-qPCR, we were unable to identify an up-regulation of the "IL-10 signaling" pathway in the peripheral blood of VL patients. On the other hand, we did identify a negative regulation of "IL-10 signaling" pathway in the transcriptional profiles of patients under remission, which might correlate with decreased levels of this cytokine and recovery after therapy [47].

We highlight that, regardless of clinical status, expression data from individuals exposed to *L*. *infantum* display positive regulations of pathways and gene modules related to "type I interferon signaling" when compared to uninfected individuals, suggesting that IFN-αβ might play important roles in infections with *L*. *infantum*. Although the role of IFN-γ in infections with *Leishmania* has been extensively explored, the function of type I interferon signaling is not clear [48]. Of note, transcriptomic profiling of lesions from patients infected with *L*. *braziliensis* identified a positive regulation of type I interferon signaling [22], while *L*. *major* induces a type I transcriptional signature in human dendritic cells [37], indicating common responses from both cutaneous and visceral infections with *Leishmania*. Of interest, our analyses suggest that the response induced by IFN-αβ signaling might depend on the context and clinical status of infection with *L*. *infantum*. In other words, while type I interferon signaling is elicited in both VL patients and asymptomatic individuals, it might present differential regulation of its transcriptional program in these two states of infection. In line with this concept, other work showed that only low doses of IFN-β protected BALB/c mice from progressive cutaneous disease [49]. Furthermore, the "type I interferon signaling" gene module identified herein is composed by some interferon regulatory factors (IRF), in which IRF-7 was shown to exhibit a crucial role for parasite control in mice infected with *L*. *donovani* [50]. Thus, a fine regulation of IFN-αβ expression and of the transcriptional programs induced by those cytokines could promote a protective response in asymptomatically infected individuals. In contrast, an unbalanced signaling by these cytokines during chronic VL can play a similar pathological role as that observed in infections with *Mycobacterium tuberculosis* and *Plasmodium* [14,51]. Indeed, chronic exposure to type I interferon could impact homeostasis of CD4^+^ T cells [52] or even counter-regulate signaling by IL-1β [53] and limit protective mechanisms against infections with *Leishmania* [54]. In addition, IFNAR-deficient mice present enhanced immunity against *L*. *amazonensis*, which correlated with a critical role of neutrophils in parasite clearance [55]. The reason for such differences between VL patients and asymptomatic individuals might depend on several factors that include the genetic background of strains of both host and parasite, host nutritional status, history of exposure to vectors, co-infections with other pathogens, among other factors known to influence the magnitude and regulation of the immune response.

Although the expression data of VL patients seems to depict a general suppression of pathways and gene modules associated with innate immune response, a decrease in proportions neutrophils and monocytes was suggested by gene expression modular analyses (Figs 3 and 4) and validated with cell decovolution analysis (Fig 5), prompting careful interpretation. Indeed, neutropenia has been shown to be an independent risk factor for death in children with VL [56], indicating that the low proportion of circulating granulocytes and monocytes translate into the down-regulation of genes coding for chemokine receptors and chemokines as *CCR1*, *CXCR1*, *CXCL8* or even neutrophil activation receptors as *FPR1*, *C5AR1*; in contrast, the up-regulation of *IFNG* underscores the increased levels of IFN-γ present in serum from VL patients [8], whereas up-regulation of both *IFNG* and *CXCL10* support the activation of T lymphocytes and development of a Th1 response during active disease [6]. Accordingly, we were unable to identify significant differences in the relative proportions of T lymphocytes from VL patients compared to other groups (Fig 5B), indicating that DEGs annotated into processes as TCR signaling were not influenced by the relative proportion of those cells.

Dysfunctional responses during chronic VL might originate from failures in proper antigen presentation and stimulation, indicated by the significant associations between polymorphisms in the HLA class II region and susceptibility to visceral leishmaniasis [57], as well as by the negative regulation of gene modules related to major histocompatibility complex (MHC) class II identified in expression data from VL patients (Fig 4C). Yet, the extensive up-regulation of pathways and gene modules related to TCR signaling and antigen presentation through MHC class I suggests a chronic stimulation of CD8^+^ T lymphocytes during VL and correlates with previous findings from studies that used different analytical approaches [58,59]. Persistent cross-linking of TCR and MHC class I without appropriate co-stimulation of CD8^+^ T lymphocytes results in an exhausted cellular phenotype, which is characterized by the expression of the inhibitory receptors PD-1, CTLA-4, LAG3, TIM3 and TIGIT [60]. Indeed, CD8^+^ T lymphocytes isolated from VL patients exhibit increased expression of inhibitory surface receptors [58]. We also identified increased expression of PD-1, CTLA-4 and LAG3 in expression data from VL patients, while expression of such genes was down-regulated in patients under remission of the disease (S1 Data). Those findings suggest that perturbations in antigen presentation pathways may lead to inefficient activation and differentiation of CD4^+^ T lymphocytes, promote the exhaustion of CD8^+^ T lymphocytes and account for parasite evasion from the host response during VL.

Proper antigen presentation is crucial for T cell activation and differentiation, but other factors might impact lymphocyte function during VL. Indeed, T cell-specific deletion of Notch 1 and Notch 2 demonstrated that they are required for efficient development of Th1 immune responses and resistance in mice infected with *L*. *major* [61], thus polymorphisms affecting such molecules and transcriptional programs induced by their activation may influence T cell responses during infections with *L*. *infantum*. Indeed, a genome-wide association study in mixed-breed dogs with VL identified a marker located between two predicted transcription factor binding sites that regulate the expression of *TLE1*, a molecule involved in the Notch signaling pathway [62]. Another perspective is given by the demonstration that Notch 1 signaling pathway drives the activation of mouse macrophages into a M1 phenotype through metabolic up-regulation of mitochondrial oxidative phosphorylation and attendant reactive oxygen species [63], molecules that are implicated in the killing of *L*. *braziliensis* by human classical monocytes [64]. In the light of those findings, the positive associations between "Notch signaling pathway" with transcriptional profiles of patients under remission of disease and asymptomatic individuals support a protective role of this pathway in human infections with *L*. *infantum*.

Cell deconvolution analysis corroborates previous investigations focused on lymphocyte proportions in peripheral blood of VL patients [45,65], whereby strong associations between transcriptional profiles of patients under remission with B lymphocyte-related modules likely reflect higher abundance of those cells in the peripheral blood after therapy [65]. Despite this, hypergammaglobulinemia is frequently observed in VL patients due to polyclonal activation of B lymphocytes [66]. However, modules related to B lymphocytes were mainly down-regulated in VL patients and asymptomatic individuals, which suggests that after infections with *L*. *infantum*, activated B lymphocytes undergo differentiation to plasma cells and migrate to specific niches such as the bone marrow [67], while those remaining in the periphery might display unique transcriptional programs and functions. Indeed, follicular T cell-mediated regulation of the B lymphocyte compartment may account for beneficial or pathogenic responses during distinct states of infection with *L*. *infantum* [68], a hypothesis that is supported by significant differences in structural and functional features of immunoglobulin G isolated from VL patients and asymptomatic individuals [8].

In conclusion, this is the first attempt to screen for blood transcriptional signatures from distinct states of infection of humans with *L*. *infantum*. Future studies including individuals of other populations, as well as investigations focused on specific pathways highlighted by these signatures shall confirm and extend the hypotheses discussed herein. These signatures point to novel directions for studying human immune responses after infections with *L*. *infantum*, which can guide development of new strategies of intervention.

## Supporting Information

S1 Fig "Antigen presentation by MHC class I" pathway map generated with GeneGO Metacore. Colored bars indicate differentially expressed genes of VL patients compared to uninfected controls, where red indicates up-regulated genes and blue indicates down-regulated genes. See MetaCore website for detailed legend at https://portal.genego.com/legends/MetaCoreQuickReferenceGuide.pdf.
(TIF)

S2 Fig "Integrin inside-out signaling in neutrophils" pathway map generated with GeneGO Metacore. Colored bars indicate differentially expressed genes of VL patients compared to uninfected controls, where red indicates up-regulated genes and blue indicates down-regulated genes. See MetaCore website for detailed legend at https://portal.genego.com/legends/MetaCoreQuickReferenceGuide.pdf.
(TIF)

S3 Fig Network visualization of co-expression gene modules identified by WGCNA. (TIF)

S4 Fig Hierarchical clustering of correlations between BTMs. For each BTM significantly enriched for distinct states of infection of humans with *Leishmania infantum*, PC1 scores were retrieved from principal component analysis and thus subjected to Pearson correlation analysis. Resulting coefficients were clustered with Euclidian distance method and ward linkage algorithm.
(TIF)

S1 Data Differentially expressed genes between states of infection with L. infantum. (XLSX)

S2 Data Co-expression gene modules identified by WGCNA. (XLSX)

S3 Data Code file for pre-processing, differential expression, WGCNA and cell deconvolution analyses. (TXT)

S1 Table Primers used in Quantitative Real Time-PCR analysis. F—forward; R—reverse.
(DOCX)

S1 Checklist STROBE Checklist. (DOC)
