# Supplementary figures and images for: Blood Transcriptional Profiling Reveals Immunological Signatures of Distinct States of Infection of Humans with Leishmania infantum
Source: PLoS Negl Trop Dis. 2016 Nov 9;10(11):e0005123. doi: 10.1371/journal.pntd.0005123 (PMC5102635; doi:10.1371/journal.pntd.0005123)

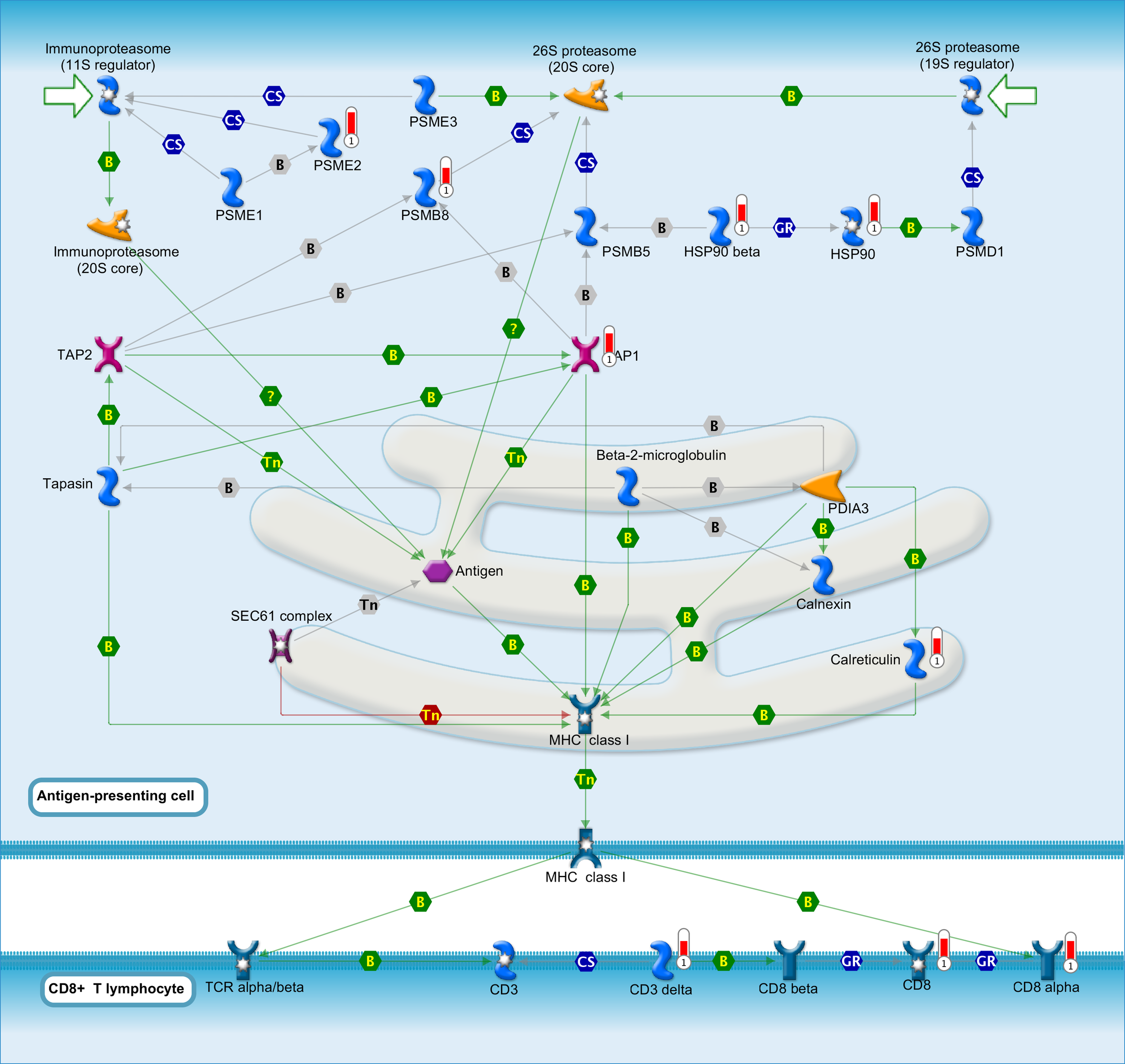

Supplement: S1 Fig — Colored bars indicate differentially expressed genes of VL patients compared to uninfected controls, where red indicates up-regulated genes and blue indicates down-regulated genes. See MetaCore website for detailed legend at https://portal.genego.com/legends/MetaCoreQuickReferenceGuide.pdf. (TIF) [file pntd.0005123.s001.tif]

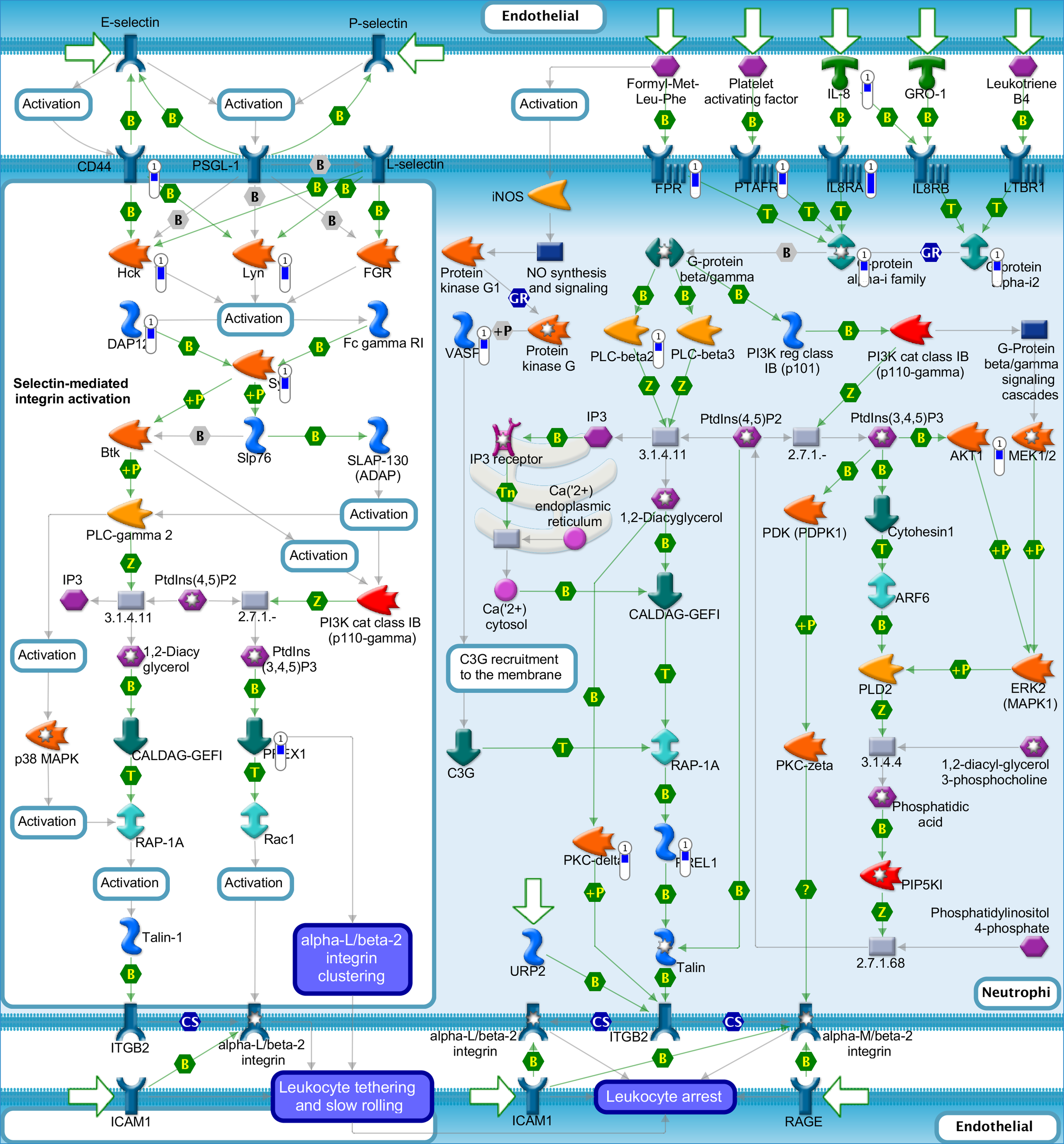

Supplement: S2 Fig — Colored bars indicate differentially expressed genes of VL patients compared to uninfected controls, where red indicates up-regulated genes and blue indicates down-regulated genes. See MetaCore website for detailed legend at https://portal.genego.com/legends/MetaCoreQuickReferenceGuide.pdf. (TIF) [file pntd.0005123.s002.tif]

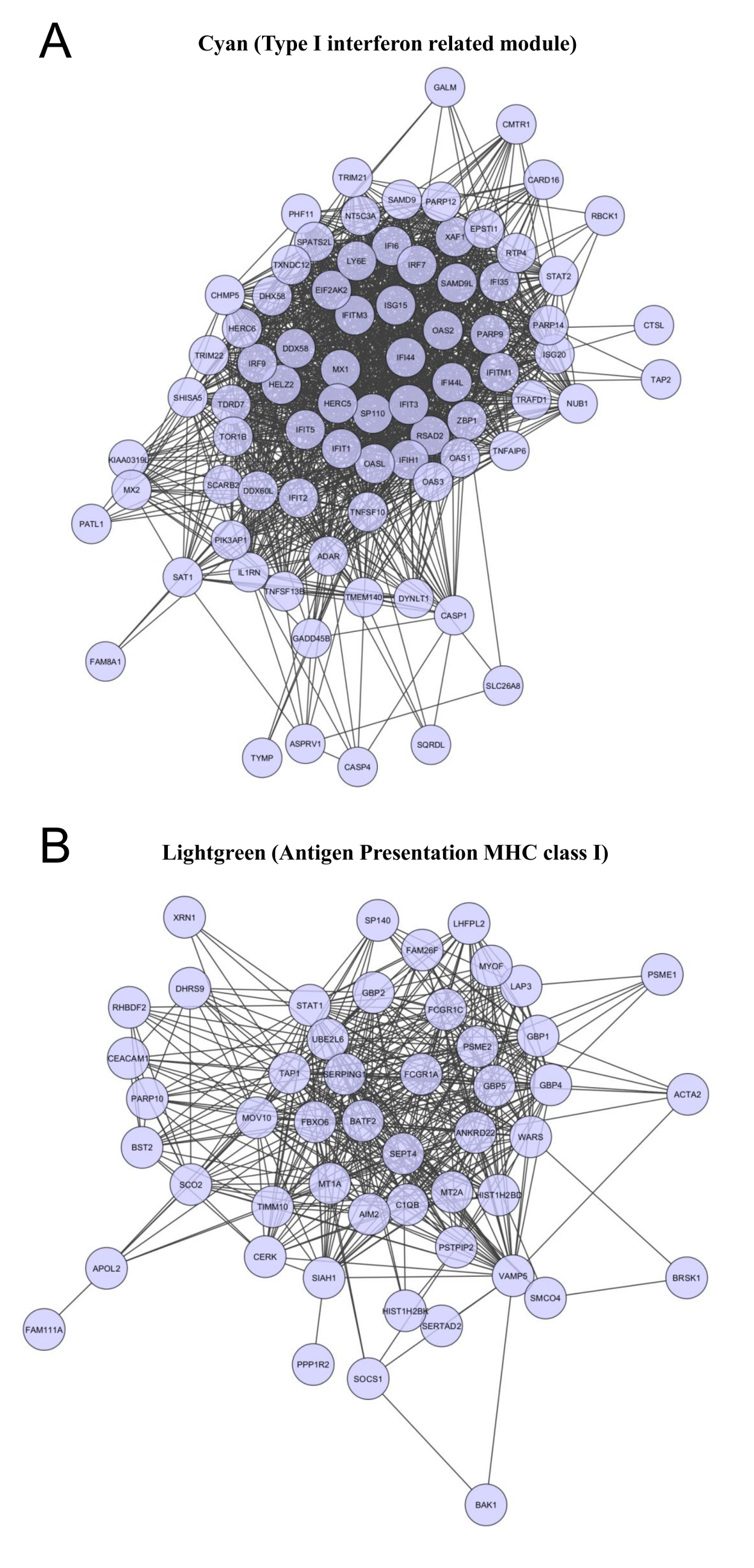

Supplement: S3 Fig — (TIF) [file pntd.0005123.s003.tif]

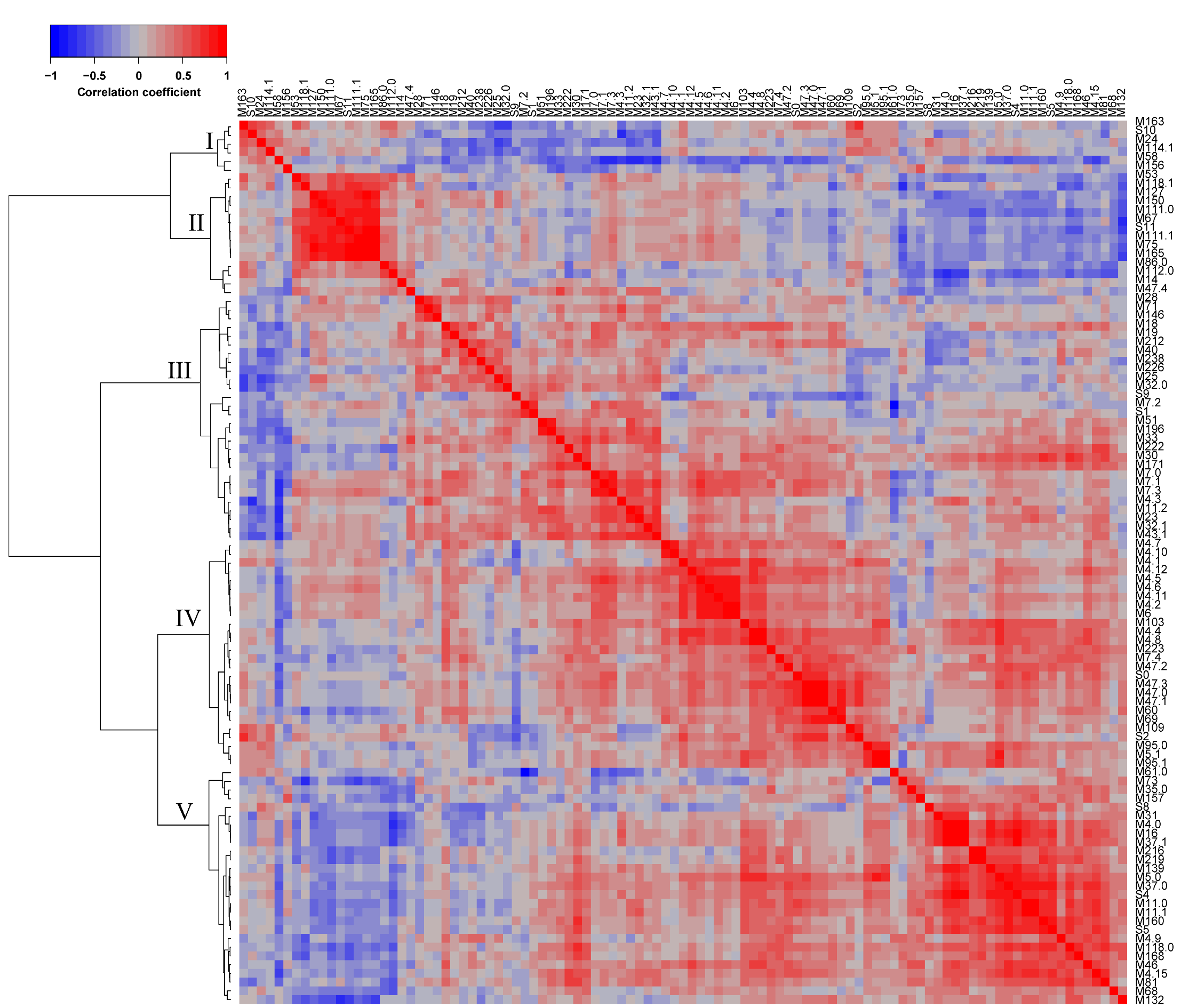

Supplement: S4 Fig — For each BTM significantly enriched for distinct states of infection of humans with Leishmania infantum, PC1 scores were retrieved from principal component analysis and thus subjected to Pearson correlation analysis. Resulting coefficients were clustered with Euclidian distance method and ward linkage algorithm. (TIF) [file pntd.0005123.s004.tif]
