# Supplementary material for: Blood Transcriptional Profiling Reveals Immunological Signatures of Distinct States of Infection of Humans with Leishmania infantum
Source: PLoS Negl Trop Dis. 2016 Nov 9;10(11):e0005123. doi: 10.1371/journal.pntd.0005123 (PMC5102635; doi:10.1371/journal.pntd.0005123)
Supplement: S1 Table — F—forward; R—reverse. (DOCX) [file pntd.0005123.s008.docx]

| **GenBank ID** | **Gene name** | **Symbol** | **Primers** |
| --- | --- | --- | --- |
| NM_005532.4 | Interferon alpha-inducible protein 27 | IFI27 | F: TGCTCTCACCTCATCAGCAGT |
|  |  |  | R: CACAACTCCTCCAATCACAACT |
| NM_001548.4 | Interferon-induced protein with tetratricopeptide repeats 1 | IFIT1 | F: GCGCTGGGTATGCGATCTC |
|  |  |  | R: CAGCCTGCCTTAGGGGAAG |
| NM_005533.4 | Interferon-induced protein 35 | IFI35 | F: GTGGACGTTCGGGAGCTAC |
|  |  |  | R: ACTGGCCGATTTGGCACAG |
| NM_000619.2 | Interferon gamma | IFNG | F: TCGGTAACTGACTTGAATGTCCA |
|  |  |  | R: TCGCTTCCCTGTTTTAGCTGC |
| NM_007315.3 | Signal transducer and activator of transcription 1 | STAT1 | F: CGGCTGAATTTCGGCACCT |
|  |  |  | R: CAGTAACGATGAGAGGACCCT |
| NM_005419.3 | Signal transducer and activator of transcription 2 | STAT2 | F: CTGCTAGGCCGATTAACTACCC |
|  |  |  | R: TCTGATGCAGGCTTTTTGCTG |
| NM_001565.3 | Chemokine (C-X-C motif) ligand 10 | CXCL10 | F: GTGGCATTCAAGGAGTACCTC |
|  |  |  | R: TGATGGCCTTCGATTCTGGATT |
| NM_000634.2 | Chemokine (C-X-C motif) receptor 1 | CXCR1 | F: CTGACCCAGAAGCGTCACTTG |
|  |  |  | R: CCAGGACCTCATAGCAAACTG |
| NM_178329.2 | Chemokine (C-C motif) receptor 3 | CCR3 | F: TGGCATGTGTAAGCTCCTCTC |
|  |  |  | R: CCTGTCGATTGTCAGCAGGATTA |
| NM_000572.2 | Interleukin 10 | IL10 | F: GACTTTAAGGGTTACCTGGGTTG |
|  |  |  | R: TCACATGCGCCTTGATGTCTG |
| NM_000566.3 | Fc gamma receptor Ia | FCGR1A | F: AGCTGTGAAACAAAGTTGCTCT |
|  |  |  | R: GGTCTTGCTGCCCATGTAGA |
| NM_021642.3 | Fc gamma receptor IIa | FCGR2A | F: GCTTCAACCATTGACAGTTTTGC |
|  |  |  | R: CCACGGGGGCTCAAGTTTC |
| NM_000591.3 | CD14 molecule | CD14 | F: CAACCTAGAGCCGTTTCTAAAGC |
|  |  |  | R: GCGCCTACCAGTAGCTGAG |
| NM_000578.3 | Solute carrier family 11 (proton-coupled divalent metal ion transporter, nramp1) | SLC11A1 | F: CTTCAGCCTGCGGAAGCTAT |
|  |  |  | R: TCTGACTCGATGTTTCCTGGG |
| NR_003286.2 | RNA, 18S ribosomal 5 | RNA18S5 | F: GTAACCCGTTGAACCCCATT |
|  |  |  | R: CCATCCAATCGGTAGTAGCG |
| NM_001101.3 | Beta actin | ACTB | F: AGGCACCAGGGCGTGAT |
|  |  |  | R: GCCCACATAGGAATCCTTCTGAC |
| NM_004048.2 | Beta-2-microglobulin | B2M | F: GCTCGCGCTACTCTCTCTTT |
|  |  |  | R: CTCTGCTGGATGACGTGAGT |
| NM_021130.4 | Peptidylprolyl isomerase A (cyclophilin A) | PPIA | F: GCACTGCCAAGACTGAG |
|  |  |  | R: CCTGCAATCCAGCTAGG |
