# Supplementary material for: Blood Transcriptional Profiling Reveals Immunological Signatures of Distinct States of Infection of Humans with Leishmania infantum
Source: PLoS Negl Trop Dis. 2016 Nov 9;10(11):e0005123. doi: 10.1371/journal.pntd.0005123 (PMC5102635; doi:10.1371/journal.pntd.0005123)
Supplement: S1 Checklist — (DOC) [file pntd.0005123.s009.doc]

STROBE Statement—Checklist of items that should be included in reports of ***case-control studies***

|  | Item No | Recommendation |
| --- | --- | --- |
| **Title and abstract** | 1 | (*a*) Blood Transcriptional Profiling Reveals Immunological Signatures of Distinct States of Infection of Humans with *Leishmania infantum* |
|  |  | (*b*) Visceral leishmaniasis (VL) can be lethal if untreated; however, the majority of human infections with the etiological agents are asymptomatic. Using Illumina Bead Chip microarray technology, we investigated the patterns of gene expression in blood of active VL patients, asymptomatic infected individuals, patients under remission of VL and controls. Computational analyses based on differential gene expression, gene set enrichment, weighted gene co-expression networks and cell deconvolution generated data demonstrating discriminative transcriptional signatures. VL patients exhibited transcriptional profiles associated with pathways and gene modules reflecting activation of T lymphocytes via MHC class I and type I interferon signaling, as well as an overall down regulation of pathways and gene modules related to myeloid cells, mainly due to differences in the relative proportions of monocytes and neutrophils. Patients under remission of VL presented heterogeneous transcriptional profiles associated with activation of T lymphocytes via MHC class I, type I interferon signaling and cell cycle and, importantly, transcriptional activity correlated with activation of Notch signaling pathway and gene modules that reflected increased proportions of B cells after treatment of disease. Asymptomatic and uninfected individuals presented similar gene expression profiles, nevertheless, asymptomatic individuals exhibited particularities which suggest an efficient regulation of lymphocyte activation and a strong association with a type I interferon response. Of note, we validated a set of target genes by RT-qPCR and demonstrate the robustness of expression data acquired by microarray analysis. In conclusion, this study profiles the immune response during distinct states of infection of humans with Leishmania infantum with a novel strategy that indicates the molecular pathways that contribute to the progression of the disease, while also providing insights into transcriptional activity that can drive protective mechanisms. |
| Introduction | | |
| Background/rationale | 2 | Infections with the protozoan parasites Leishmania donovani or L. infantum (chagasi) result in clinical outcomes that range from asymptomatic infection to active visceral leishmaniasis (VL). When disease occurs, symptoms often include fever, hepatosplenomegaly, cachexia, pancytopenia and hypergammaglobulinemia (1), while the lethality of VL correlates with severe symptoms such as secondary infections, hemorrhage, liver failure and cardiotoxicity due to treatment (2). Depressed cellular immunity is considered a hallmark of VL, which is evidenced by the inability of VL patients to develop a positive delayed type hypersensitivity (DTH) in Montenegro skin tests in spite of infection (3), and the absence of IFN-γ in cultures of peripheral blood mononuclear cells stimulated with leishmanial antigens (4). On the other hand, whole blood assays showed that VL patients do not lack the ability to mount Leishmania specific IFN-γ responses (5). Furthermore, peripheral blood or splenic CD4+ T lymphocytes from VL patients produce IFN-γ in response to leishmanial antigens, which is also crucial to limit parasite replication in splenic aspirate cultures (6). These findings indicate that progression of VL involves other molecular mechanisms besides failures in activation and differentiation of CD4+ T lymphocytes. Development and severity of VL have been associated with several pro-inflammatory and immunoregulatory factors such as cytokines (7,8), lipopolysaccharide (9), mannan-binding lectin (10), C reactive protein and patterns of IgG Fc N-glycosylation (8). In addition, studies addressing features of infected asymptomatic individuals point towards a fine regulation of several immune compartments thought to control parasites without damage to the host (8,11,12). Thus, particular clinical outcomes after infections with L. infantum are influenced by complex multi-factorial immunological processes. Re-circulation between central and peripheral lymphoid organs has a major impact on effective immune responses and infections and inflammation cause cell migration via lymphatic and circulatory systems (13). During physiological or pathological events in which factors are released systemically, features of peripheral cell re-circulation provide an informative platform to study the human immune system with molecular methods of genomic scale, which have been used to investigate blood transcriptional and immunological profiles during human infections, including parasitic diseases (14–16). Genome-wide profiling strategies have been employed to evaluate in vitro systems of infection with Leishmania and in vivo models of VL (17–20), while studies in humans are limited to biopsies from patients with cutaneous leishmaniasis (21,22). |
| Objectives | 3 | Gardinassi et al. hypothesized that a global overview of gene expression in the peripheral blood of humans presenting with distinct states of infection with L. infantum could reveal unappreciated immunological features that account for pathological or protective responses. To address this issue, we undertook a series of molecular approaches and functional analyses to uncover the transcriptional activity of the immune response that extend the understanding and provide new insights into the immunobiology of human VL. |
| Methods | | |
| Study design | 4 | Case-control study to identify immunological signatures of infections with *Leishmania infantum* using blood transcriptomics |
| Setting | 5 | The collection of samples took place in the Teresina, Brazil between May - August 2013. |
| Participants | 6 | (*a*) The study was conducted as per protocols approved by the Research Ethics Committee of the Clinics Hospital of the Ribeirão Preto Medical School - USP (protocol 2347/2012). All the methods were carried out in accordance with approved guidelines. Informed written consent was obtained from all of the participants or their parents or legal guardians. Whole peripheral blood was collected from patients with symptoms of VL admitted to Natan Portella Institute of Tropical Diseases, UFPI, Teresina-PI, Brazil. Diagnosis was confirmed by identification of Leishmania amastigotes in Giemsa-stained smears of bone marrow aspirate, and patients diagnosed with VL received treatment according to Brazilian guidelines (23). Additionally, whole peripheral blood was collected from a distinct group of VL patients at 2 to 5 months after the beginning of therapy with pentavalent antimonial, which were under remission of the disease (Table 1). Study subjects also included healthy individuals living in the same areas and considered to be asymptomatically infected with L. infantum, who were identified by a positive delayed type hypersensitivity (DTH) to leishmanial antigens (Table 1). Controls included individuals from different regions of Brazil (Teresina-PI and Ribeirão Preto-SP) who presented a negative DTH to leishmanial antigens (Table 1). |
| Variables | 7 | Demographic data (self reported age and sex) were recorded for each subject. Whole blood cell counts, red blood cell counts, levels of haemoglobin and hematocrit were recorded for VL patients and patients under remission of disease. |
| Data sources/ measurement | 8* | Whole peripheral blood was collected. Isolation and purification of total RNA was performed using the PAXgene Blood RNA Kit (PreAnalytix) according to the manufacturer’s instructions. RNA concentration was verified with NanoDrop 1000 spectrophotometer (NanoDrop Technologies, Wilmington, DE, USA) and the RNA integrity was determined using an Agilent 2100 Bioanalyzer (Agilent Technologies, Foster City, CA, USA). The RNA samples were submitted to microarray hybridization at the Functional Genomics Unit of the Roy J. Carver Biotechnology Center, University of Illinois, Urbana-Champaign, Illinois, USA. All procedures were performed according to the manufacturer’s instructions. Briefly, cRNA amplification and labelling was carried out on 1 ug of total RNA by using an Illumina TotalPrep Amplification kit (Ambion, Austin, TX, USA). The samples were then hybridized onto on Illumina HumanHT-12 v4 Expression BeadChips that were scanned with an Illumina iScan System (Illumina, San Diego, CA, USA). Illumina´s Beadstudio software was used to generate signal intensity values from the scans. Raw data were processed using the R Language and Environment for Statistical Computing (R) 3.2.0 (24) in association with Bioconductor 3.1 (25). The lumi package for R (26) was used to perform quality control, log2 transformation and normalization with robust spline normalization (RSN) method. This processing pipeline was based on the comparison and variation of transformation and normalization methods and optimized according to the number of samples, as well as the array technology (27). Data was filtered to remove unexpressed genes based on detection call p-values computed for each probeset of the > 47,000 probes present on the Illumina HumanHT-12 v4 array and 17,015 probes were retained for further analysis. Probe-level expression data files were deposited at the Gene Expression Omnibus (GEO) repository under accession number GSE77528. |
| Bias | 9 | For asymptomatic individuals and uninfected individuals, we collected samples before performing delayed type hypersensitivity tests, those who were positive were classified as asymptomatic and those who were negative as uninfected. |
| Study size | 10 | The study size was designed according to previous publications. |
| Quantitative variables | 11 | Quantitative analyses from microarray data were compared between groups to identify distinct transcriptional signatures reflecting the immune response for each group. |
| Statistical methods | 12 | (*a*) The patterns of differential gene expression between the study groups were evaluated by generating linear models and moderated *t*-statistic or ANOVA with the package Limma for R (28). *P* values were adjusted with Benjamini-Hochberg false discovery rate (FDR) correction. Hierarchical clustering of expression data performed with Euclidian distance and complete algorithm linkage. For WGCNA, Log-transformed, normalized expression data were filtered by the 3700 most variant genes. A soft threshold power beta was chosen based on the scale-free topology criterion (31). Constructed gene networks were then used to identify modules from the topological overlap matrix. |
| Results | | |
| Participants | 13* | (a) The study was composed by 45 participants. |
| Descriptive data | 14* | (a) Demographic data (self reported age and sex) were recorded for each subject. Whole blood cell counts, red blood cell counts, levels of haemoglobin and hematocrit were recorded for VL patients and patients under remission of disease. |
| Outcome data | 15* | VL patients (n = 8), patients under remission (n = 8), asymptomatic individuals (n = 14) and uninfected controls (n = 15). |
| Main results | 16 | (*a*) Give unadjusted estimates and, if applicable, confounder-adjusted estimates and their precision (eg, 95% confidence interval). Make clear which confounders were adjusted for and why they were included – NOT APPLICABLE |
|  |  | (*b*) Report category boundaries when continuous variables were categorized – NOT APPLICABLE |
|  |  | (*c*) If relevant, consider translating estimates of relative risk into absolute risk for a meaningful time period – NOT APPLICABLE |

| Other analyses | 17 | Using Illumina Bead Chip microarray technology, we investigated the patterns of gene expression in blood of active VL patients, asymptomatic infected individuals, patients under remission of VL and controls. Computational analyses based on differential gene expression, gene set enrichment, weighted gene co-expression networks and cell deconvolution generated data demonstrating discriminative transcriptional signatures. |
| --- | --- | --- |
| Discussion | | |
| Key results | 18 | The mechanisms that drive progression towards disease or protect individuals from developing symptoms while infected with Leishmania parasites remain poorly understood. Using a genome-wide approach to investigate patterns of gene expression from whole blood, we identified transcriptional profiles that shed light on pathways and/or gene expression modules associated with distinct states of human infections with L. infantum. |
| Limitations | 19 | Transcriptional signatures from human samples have been shown to be sensitive to factors such as age and sex (38) and sample size (39). The groups evaluated in this study did not exhibit significant differences in distribution of age or sex. Similar numbers of patients infected with L. braziliensis and controls were evaluated by pioneering studies that not only identified unique transcriptional signatures, but were also able to recapitulate previously described immunopathological responses in lesions of individuals with cutaneous leishmaniasis (21,22,40). Of note, samples from patients under remission of disease were collected during distinct time points after the beginning of therapy, which could influence their blood transcriptional profiles. Nonetheless, we were able to identify transcriptional signatures that segregated patients recovering from disease from VL patients before therapy (Figure 1C); concomitantly, relative to asymptomatic individuals or uninfected controls, the majority of patients under remission of disease exhibited strong correlations of levels of expression for DEGs (Figure 1C). Furthermore, linear model-based statistical analysis and adjusted P values (FDR) did not detect significant differences between the transcriptional profiles of asymptomatic individuals and uninfected controls (Figures 1B and 1C). However, uncorrected P values retrieved 620 differentially expressed probes (S1 Data), which included probes for genes shown to be differentially expressed by RT-qPCR (Figure 6). These results demonstrate that, compared to uninfected controls, asymptomatic individuals present only a subset of differentially expressed genes, whereby the large number of comparisons between 17,105 probes (12,491 genes) can lead to a type II statistical error and inflate the rate of false negatives (41). To overcome this issue, we conducted distinct approaches with the ability to estimate the differences between the transcriptional profiles of asymptomatic individuals and uninfected controls; the combination of distinct functional analyses and common features retrieved by them support the robustness of the immunological signatures identified for distinct states of infections with L. infantum. |
| Interpretation | 20 | It is noteworthy that the transcriptional signatures identified in this study discriminated between VL patients from patients under remission of disease and healthy individuals (Figure 1C). Importantly, by assessing the levels of expressions of a set of target genes by RT-qPCR we validated the robustness of the expression data acquired by microarray analysis (Figure 6). Of note, samples from patients under remission of disease were collected during distinct time points after the beginning of therapy, which could influence their blood transcriptional profiles. Nonetheless, we were able to identify transcriptional signatures that segregated patients recovering from disease from VL patients before therapy (Figure 1C). linear model-based statistical analysis and adjusted P values (FDR) did not detect significant differences between the transcriptional profiles of asymptomatic individuals and uninfected controls (Figures 1B and 1C). However, uncorrected P values retrieved 620 differentially expressed probes (S1 Data), which included probes for genes shown to be differentially expressed by RT-qPCR (Figure 6). These results demonstrate that, compared to uninfected controls, asymptomatic individuals present only a subset of differentially expressed genes, whereby the large number of comparisons between 17,105 probes (12,491 genes) can lead to a type II statistical error and inflate the rate of false negatives (41). To overcome this issue, we conducted distinct approaches with the ability to estimate the differences between the transcriptional profiles of asymptomatic individuals and uninfected controls; the combination of distinct functional analyses and common features retrieved by them support the robustness of the immunological signatures identified for distinct states of infections with L. infantum. |
| Generalisability | 21 | We propose that in-depth analysis of transcriptional profiles from populations analyzed in this study, as well as longitudinal studies including patients followed throughout treatment can be useful for the prospection of new biomarkers of VL or asymptomatic infection, as well as for the prognosis after treatment and remission of disease. |
| Other information | | |
| Funding | 22 | This work was supported by the Fundação de Amparo à Pesquisa do Estado de São Paulo- FAPESP (grant number 12/06708-2 to IKFMS) and the Conselho Nacional de Desenvolvimento Científico e Tecnológico (grant number 467608/2014-8 to IKFMS). LGG and GRG received scholarships from FAPESP (2011/23819-0 and 2014/25856-8; 2013/00382-0, respectively). |

*Give information separately for cases and controls.

**Note:** An Explanation and Elaboration article discusses each checklist item and gives methodological background and published examples of transparent reporting. The STROBE checklist is best used in conjunction with this article (freely available on the Web sites of PLoS Medicine at http://www.plosmedicine.org/, Annals of Internal Medicine at http://www.annals.org/, and Epidemiology at http://www.epidem.com/). Information on the STROBE Initiative is available at http://www.strobe-statement.org.
